# Preventive effects of probiotics on dental caries in vitro and in vivo

**DOI:** 10.1186/s12903-024-04703-x

**Published:** 2024-08-08

**Authors:** Jingyan Zhang, Qian Wang, Zhi Duan

**Affiliations:** Nutrition & Health Technology Center, Qingdao Vland Biotech Group Co., Ltd, Qingdao, China

**Keywords:** Dental caries, *S. mutans*, Caries models, Probiotics

## Abstract

**Background:**

Dental caries is a common disease in the oral cavity, and the microorganisms in the cavity are colonized in the form of dental plaque biofilm. *Streptococcus mutans* is the main pathogen causing dental caries. Using probiotics to inhibit the growth and colonization of pathogenic bacteria, regulate mucosal immunity and improve oral microecological balance is an effective way to prevent or treat dental caries. The aim of this study was to evaluate the caries-prevention of probiotics in vitro and in rat caries models.

**Methods:**

The probiotics used in this study are a combination of 4 strains of bacteria. After the fermentation of 4 strains (*L. plantarum*, *L. salivarius*, *L. rhamnosus*, and *L. paracasei*) was completed, they were mixed in equal volume proportions and used as samples to be tested. The mixture was then assessed the ability to inhibit the growth of *S. mutans* in vitro and in vivo. SPSS Statistics 22.0 (SPSS, Inc., Chicago, IL, USA) was used for analysis.

**Results:**

In vitro the probiotics mixture could inhibit the growth of *S. mutans* and was able to remove biofilms formed by *S. mutans*. In a 42-day in vivo experiment, the probiotics group significantly reduced the level of *S. mutans* on the tooth surface of rats, reducing more than half the bacterial quantities compared with the caries model group (*P* < 0.05). The amount of *S. mutans* in the antagonist group was low and highly significant compared with the caries model group. Moreover, the mixture of 4 strains significantly reduced the caries scores (modified Keyes scoring method) in both the probiotic and antagonist groups (*p* < 0.05).

**Conclusions:**

The study showed that the combination of the four strains can reduce the cavity scores, and the four strains can be used as products in oral care products. At the same time, the study also suggests that probiotic therapy can be an effective way to prevent dental caries.

## Background

Dental caries is one of the most common oral diseases worldwide, particularly in developing countries [1]. The WHO Global Oral Health Status Report (2022) estimated that oral diseases affect nearly 3.5 billion people worldwide, with 3 out of 4 people living in middle-income countries [2]. Globally, an estimated 2 billion people suffer from caries of permanent teeth, and 514 million children suffer from caries of primary teeth. In 2016, caries in permanent and deciduous teeth ranked second and fifth, respectively, among the 10 diseases with the highest global incidence. Caries is the result of the complex interaction between dietary carbohydrates and cariogenic microorganisms in oral bioflms, also influenced by the quality and quantity of saliva; the disease results from shifts in the balance of the resident microbiota driven by changes in the local environmental conditions [3]. Recent studies have shown that tooth decay is the result of a synergistic action of several species. Of these, *S. mutans*, *L. acidophilus*, and *Actinomyces viscosus* may be considered the main pathogenic species involved in the initiation and development of dental caries [4]. *Streptococcus mutans* is one of the main microbial pathogens of dental caries. [5]. The cell surface protein PAc is an *S. mutans* virulence factor because it is involved in the initial adherence of the organism to tooth surfaces [6, 7]. As *S. mutans* adheres to the tooth surface, it forms biofilm dental plaque. The formation of a bacterial biofilm plaque is a prerequisite for the occurrence of caries [8]. Many studies have reported the relationship between the presence of *S. mutans*, biofilm formation, and the related risk of caries [9–11]. Therefore, inhibiting the growth of *S. mutans* and removing the biofilm formed by *S. mutans* is an important method of treating dental caries.

Once dental plaque has formed, it can damage the structure of the tooth and even destroy the nerve causing toothache. Finding an effective way to prevent or reduce the cavity scores is important for oral health. Probiotics are a group of active microorganisms that are beneficial to their hosts [12]. Probiotics have been recognized in regulating gut health [13–15], and there also can be effective in oral health [16, 17]. For example, Chuang et al. [18] reported that probiotic *L. paracasei* GMNL-33 may reduce caries-associated salivary microbial counts in healthy adults. Ahola et al. [19] reported that short-term consumption of cheese containing *Lactobacillus rhamnosus* GG and *Lactobacillus rhamnosus* LC705 can reduce the counts of *Streptococcus mutans* and *salivary yeasts* in young people. Villavicencio et al. [20] showed that consumption of milk with *Lactobacillus rhamnosus* and *Bifidobacteruim longum* could reduce the amount of *S. mutans* in saliva. Teanpaisan et al. [21, 22] revealed that either short-term or long-term consumption of milk powder containing *L. paracasei* SD1 could reduce salivary cariogenic mutans streptococci resulting in low risk for caries. Our previous studies explored the ability of *L. plantarum* VHProbi V38 and *L. rhamnosus* VHProbi M14 to inhibit the growth of *S. mutans* and remove biofilms formed by *S. mutans*in vitro [23, 24]. The screening of candidate oral probiotics based on their abilities to inhibit *S. mutans* growth and biofilm formation may represent an alternative means of preventing tooth decay.

In this study, we first evaluated the inhibition and biofilm removal effects of the combined use of 4 strains of probiotics on *S. mutans*in vitro. Then we also examined oral *S. mutans* colonization and caries formation in rats using bacterial counts and caries scores to evaluate the effect of probiotics on the prevention or treatment of caries in vivo. These results will support and inform the development of probiotics to prevent caries.

## Methods

### Bacterial strains and mice

*S. mutans* strains BNCC 700,610 and ATCC 25,175 were purchased from the BeNa Culture Collection Centre (Beijing, China). *S. mutans* CCTCC AB 99,010 was purchased from the China Center for Type Culture Collection (Wuhan, China). The 3 strains were cultured in the brain-heart infusion (BHI) broth both separately for 24 h with a bacterial load of 10^8^ CFU/mL in aerobic conditions at 37℃ and then mixed in a volume ratio of 1:1:1. All references to *S. mutans* fluids in this article refer to a mixture of the 3 strains.

*L. plantarum* VHProbi V38, *Ligilactobacillus salivarius* VHProbi A17, *L. rhamnosus* VHProbi M14 and *L. paracasei* VHProbi OF10 were kept in China Center for Type Culture Collection (NO: M2022173, M2022172, M2022171 and M2022170, Wuhan, China). The 4 strains were individually incubated for 24 h in Man, Rogosa, and Sharpe (MRS) broth with a bacterial load of 10^8^ CFU/mL. They were then mixed in equal volumes to prepare the probiotic mixture for the test. All references to *Lactobacillus* liquid in this article refer to a mixture of the 4 strains.

Twenty 3-week-old weanling specific pathogen-free (SPF) male Wistar rats were provided by the Qinglong Mountain Animal Breeding Centre (Jiangsu, China) under license number SCXK(Su)2017-0001. The rats were fed a special pellet diet (Diet 2000, Jiangsu Synergy Biological Co., Ltd.) and housed in an SPF-class animal house after the caries model was constructed. Housing conditions included a 12 h/12 h light/dark cycle, free feeding and watering, a temperature of 20–26 °C, and relative humidity between 40% and 70%.

### Bacteriostatic activity testing

The antibacterial activities of *Lactobacillus* strains against *S. mutans* were quantified using a modified agar-diffusion method and co-culture in vitro [25–28].

A volume of 100 mL melted BHI agar medium, maintained at ~ 45℃, was inoculated with 300 µL mixed bacterial suspension of *S. mutans* (OD_600_ 0.5–0.6). The medium (7 mL) containing *S. mutans* was quickly poured into solidified agar plates. After medium solidification, Oxford cups were placed on the surfaces of the cultures, and 100 µL of the supernatant of lactic acid bacteria (LAB) isolates was dripped into the Oxford cup. All experiments were performed in triplicate. The growth inhibitory activity of the supernatant was calculated by measuring the diameter of the inhibition zone.

The BHI broth (10 mL) was initially mixed with 0.3% (v/v) of the lactic acid bacteria culture (10^8^ CFU/mL) and *S. mutans* culture (10^8^ CFU/mL) as the inoculants, and cultured at 37℃ for 48 h. The control group was inoculated with *S. mutans* in BHI broth only. Samples were taken to determine the viable bacterial counts (CFU/mL) for *S. mutans* using Mitis Salivarius Bacitracin agar. All tests were conducted in triplicate. The inhibition rate (%) was calculated as follows:

1 $${\rm{Inhibition}}\,{\rm{rate}}\,\left( {\rm{\% }} \right){\rm{ = }}\left( {{\rm{1 - count}}\,{\rm{in}}\,{\rm{the}}\,{\rm{experimental}}\,{\rm{group/count}}\,{\rm{in}}\,{\rm{the}}\,{\rm{control}}\,{\rm{group}}} \right){\rm{*100}}$$

### Biofilm elimination testing

Biofilm removal assay in polystyrene 24-well (flat bottom) plates was performed using the method by Zhang et al. [23] and Khan et al. [29]. A total of 600 µL of the *S. mutans* cultures in BHI broth was transferred to a 24-well plate with chamber slides and incubated for 24 h to form a biofilm at 37℃ under aerobic conditions. The plate was washed twice with phosphate buffer solution (PBS) (pH 7.0, 0.05 M potassium phosphate). 600 µL of cell suspension (or the supernatant) of lactic acid bacteria was inoculated into the wells. The cell suspension of lactic acid bacteria was harvested by centrifugation at 5,000 × g for 15 min, washed 3 times with PBS, and resuspended in PBS at a concentration of approximately 10^8^ CFU/mL. MRS broth alone was used as the control. After inoculation, all plates were incubated at 37℃ for 24 h to measure the ability of the samples to remove biofilms. All tests were conducted in triplicate. The culture medium was then decanted, and the plates were gently washed 3 times with 600 µL sterilized PBS to remove planktonic and loosely bound cells.

The biofilm removal rate was determined by counting the number of bacteria on the chamber slides. The chamber slides were removed and placed in sterile homogeneous bags. The slides were then subjected to a 10-minute ultrasonic wash to diffuse the organisms into the buffer solution. After a series of dilutions, 100 µL of the samples were cultured into Mitis Salivarius Bacitracin (MSB) agar plates containing 400 U/L bacitracin (G-clone, 65000u/g, China) to determine the concentration of *S. mutans*. The following formula was used to calculate the removal rate of biofilm:

2 $${\rm{Removal}}\,{\rm{rate}}\,\left( {\rm{\% }} \right){\rm{ = }}\left( {{\rm{1 - count}}\,{\rm{in}}\,{\rm{the}}\,{\rm{experimental}}\,{\rm{group/count}}\,{\rm{in}}\,{\rm{the}}\,{\rm{control}}\,{\rm{group}}} \right){\rm{*100}}$$

### Animal experiments

#### Rat caries model and general procedures

Twenty rats (3 weeks old) were allowed to adapt to the diet and environment for 3 days. They were then divided randomly into 4 groups (*n* = 5/group), comprising a normal group (N), caries control group (C), probiotic group (P), and antagonist group (A). The rats in each group were then given antibiotic water at a concentration of 200 mg/L of penicillin and 1500 mg/L of streptomycin for 3 consecutive days. On day 7, the rats were taken off antibiotic water and fasted from food and water for 2 h. Saliva was collected from each group using sterile cotton swabs. The swabs were suspended in sterile saline, and the bacterial count was detected using MSB agar. The rats were fed with sterilized chow and water. The caries model was started when the MSB plates were negative. The rats in the C and P groups were subjected to 7 consecutive days of dental caries modeling. In brief, 1 mL of *S. mutans* culture (10^8^ CFU/mL) was dipped into a sterile cotton stick. The sterile cotton swab was then applied to the rat’s oral cavity for 15 s per quadrant, as described by Beiraghi et al. [30]. On day 7, oral swabs were spread on MSB agar plates supplemented with 400 µg/L bacitracin (G-clone, 65000u/g, China) to confirm *S. mutans* colonization in the oral cavity. After successful molding, the C group was treated with high-sugar feed sterilized by Co60 irradiation and observed for 5 weeks. The P group was continuously treated with 1 mL probiotic mixture (10^8^ CFU/mL) for 1 week and observed for 4 consecutive weeks. The N group consumed a regular diet and water throughout the 6-week experimental period. The A group was treated with 2 mL mixed fermentation solution (the probiotic mixture: *S. mutans* culture = 1:1) for one week and observed for 5 consecutive weeks. During the experiment, all groups except the N group were fed 5% glucose water and a high-sugar diet sterilized by Co60 irradiation for six weeks. The ingredients of the high-sugar diet (Diet 2000 caries modelling feed) were: whole meal flour 6%, sucrose 56%, skimmed unsweetened milk powder 28%, yeast 4%, alfalfa meal 3%, liver powder 1% and salt 2%. The regular diet was rat maintenance feed (HFK 1025, Beijing, China). The experimental protocol is shown in Table 1.

This study was carried out at the laboratory of BIOGENE Biotech. Co. Ltd. (Nanjing, China), which has been approved by Laboratory Animal Ethics Committee Nanjing BIOGENE Biotech. Co. Ltd. All methods were carried out in accordance with relevant guidelines and regulations. All methods were in accordance with ARRIVE guidelines.

**Table 1:** The experimental protocol in different groups

Group	Week 0	Week 1	Week 2	Weeks 3–6
N	Days 1–3 acclimatized, Days; 4–6 Antibiotic watering.	Normal diet	Normal diet	Normal diet
C	Same as the normal group	Caries model construction	Observations	Observations
P	Same as the normal group	Caries model construction	Application of probiotic mixture	Application of probiotic mixture
A	Same as the normal group	Application of probiotic and *S. mutans* mixture	Observations	Observations

A, antagonist; C, caries control; N, normal; P, probiotic

### Observations and microbial analysis

We observed the mortality, mental activity, and hair shine of mammary rats and recorded the weight of rats every week.

The whole experiment lasted 6 weeks, during which 4 samples were collected on days 7, 14, 28, and 42. The N group was tested in the first week only. The samples were collected by rubbing a sterile cotton swab in clockwise circles on the molar occlusal surface of the mouth of each group. The swab was immediately placed in a centrifuge tube containing 1 mL of saline for 2 min. After a series of dilutions, bacterial counts were performed on the MSB plates with the appropriate dilution.

### Caries scoring and X-ray test

At the end of the 6-week trial, the rats were placed in the euthanasia chamber (Fengshi FSZZ-2 A, Suzhou, China), then the lid was fastened. A CO_2_ gas line was connected and CO_2_ was injected into the chamber at a rate of 10–30% of euthanasia box volume per min. After the rats have collapsed and lost the ability to move, the gas flow rate can be increased to a maximum of 0.5 Kpa. The CO_2_ was not switched off until the rats were immobile, not breathing and its pupils were dilated. Then the rats were observed for two minutes to determine death. The skull was removed, the soft tissues on the teeth and jaws were peeled off with a scalpel, and the jaws were cleaned with ultrasound for 20 min. Then the skull was placed in an autoclave at 121 °C for 15 min. The attached soft tissue was again peeled off with a scalpel, and the jaw was cleaned and dried at room temperature. The teeth on the left side of the rat were used to assess caries scores. The upper and lower jaws were soaked in 10% paraformaldehyde for 24 h and then washed and dried. All specimens were immersed in a 0.4% ammonium purpurate staining solution for 12 h, rinsed, and semi-sectioned along the occlusal surfaces of maxillary and mandibular molars using a diamond cutter (thickness: 0.1 mm). Caries on the rat molars were observed and evaluated under a stereomicroscope according to the caries diagnosis and scoring method reported by Keyes [31]. The method of assigning values involves judging by eye the linear extent of lesions in one plane and recording the depth of penetration under 4 headings, including enamel only (E), slight dentinal (Ds), moderate dentinal (Dm), and extensive dentinal (Dx). The scoring rules are shown in Table 2 [31].

The teeth on the right side of the rat were used for x-ray detection. Rat mandibles were scanned using a small animal X-ray machine, and the pictures were analyzed after scanning.

**Table 2:** Number of linear units assigned to each molar

Caries site	Molars					
	Mandibular			Maxillary		
	1st	2nd	3rd*	1st	2nd	3rd*
Buccal	6	4	4	6	4	3
Lingual	6	4	4	6	4	3
Sulcal	7	5	2	5	3	2
Proximal	1	2**	1	1	2**	2

*1st, 2nd, and 3rd denote the first, second, and third molars, respectively

**The second molar contains a near-middle and far-middle neighborhood

### Statistical analysis

SPSS Statistics 22.0 (SPSS, Inc., Chicago, IL, USA) was used for analysis. All data are presented as the mean ± SD. Where applicable, ANOVA or a 2-tailed Student’s t-test was used to analyze the differences between treatments. A *p*-value < 0.05 was considered statistically significant. A *p*-value < 0.01 was considered highly statistically significant.

## Results

### In vitro experiments

**Fig. 1:**
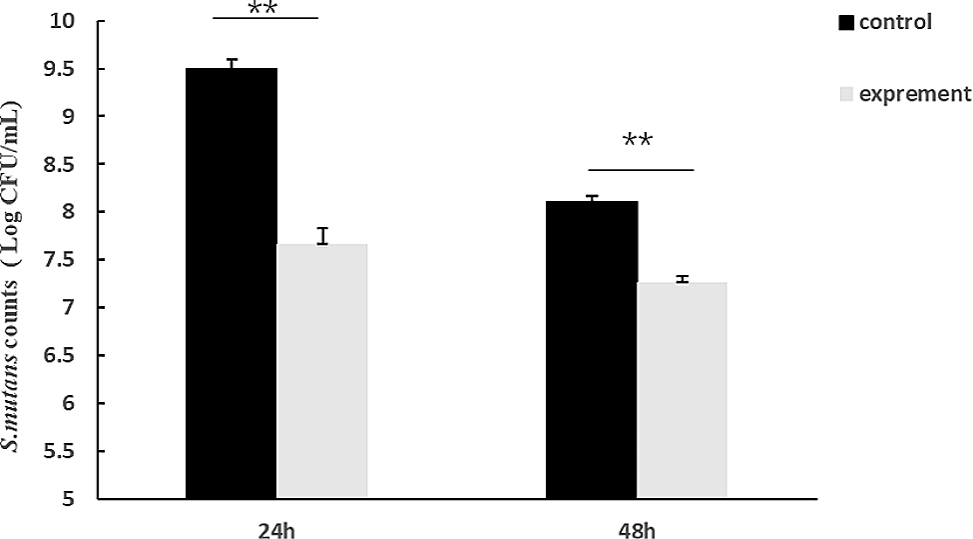
The amounts of *S. mutans* in Co-culture condition. *P < 0.05, compared with the control group; ** *p* < 0.01, compared with the control group

The inhibition diameter of the supernatant was 2.45 ± 0.05 cm. This indicates that the mixture of the four strains was able to inhibit the growth of *S. mutans* under static conditions. Under co-culture conditions, *Lactobacillus* culture was able to significantly inhibit the growth of *S. mutans*. As shown in Fig. 1, the amounts of bacteria in the control group were 9.50 ± 0.10 log CFU/mL, while the amounts of bacteria in the experimental group were 7.66 ± 0.17 log CFU/mL at 24 h. The amounts of bacteria in the control group were 8.10 ± 0.06 log CFU/mL, while the amounts of bacteria in the experimental group were 7.27 ± 0.06 log CFU/mL at 48 h, The inhibition rate at 24 h was 98.48%±0.54%. As the growth time was extended, the bacterial load of both *Lactobacillus* and *S. mutans* decreased and the inhibition rate became lower, with an inhibition rate of 84.18%±2.11%.

**Fig. 2:**
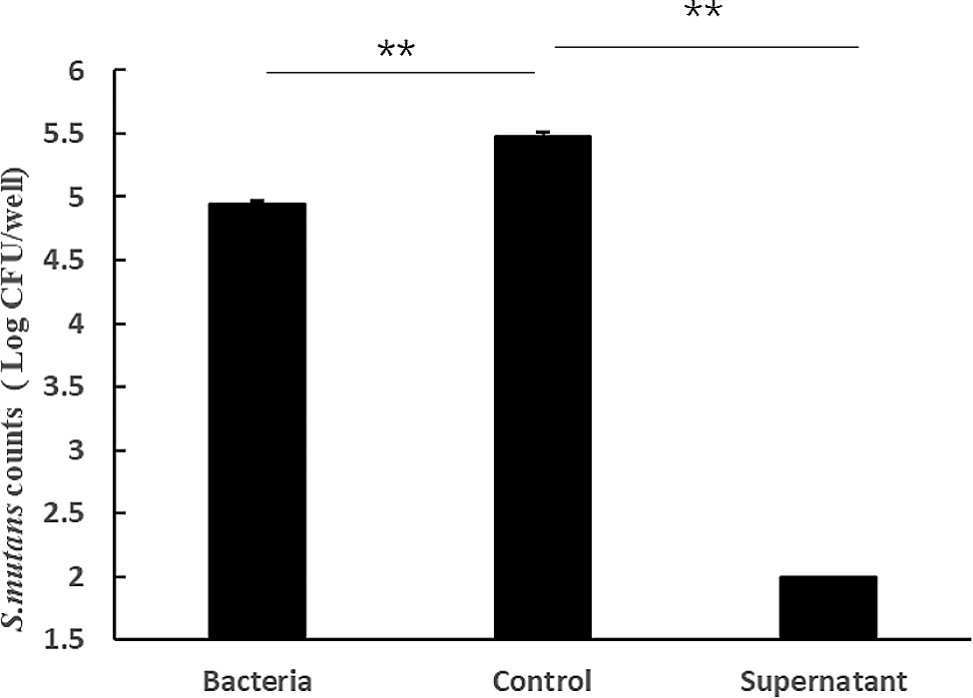
The amounts of *S. mutans* in the supernatant, the bacteria and the control groups. *P < 0.05, compared with the control group; ** *p* < 0.01, compared with the control group

The mixture of 4 strains were able to reduce the number of *S. mutans* in the biofilm as shown in Fig. 2. The number of *S. mutans* on the chamber slides of the probiotic-added group was much lower than that of the control group. In the control group, the number of *S. mutans* was 5.47 ± 0.05 log CFU/well. In the supernatant group, the number was < 2.00 ± 0.00 log CFU/well. In the bacteria group, the number was 4.95 ± 0.02 log CFU/well. The supernatant was able to completely remove the biofilm of *S. mutans* with 100%±0.00% removal, while the bacteria was able to partially remove the biofilm with 68.45%±0.74% removal.

### Animal experiments

#### General status of WISTAR rats in each group

During the entire experimental period, the rats in each group had normal activity, shiny hair, pelleted feces, and no diarrhea. The body weight of each group of rats is shown in Table 3. No inflammation, erythema, or swelling of the oral cavity was observed in any group of rats after *S. mutans* application. There was no mortality in any of the groups throughout the observation period, and there was no significant difference in weight gain between the N group and the other groups (*p* > 0.05).

**Table 3:** Results of body weight (g) recording for each group of rats

Group	0d	7d	14d	28d	35d	42d
Normal	56.9 ± 0.7	88.4 ± 3.0	133.1 ± 6.2	180.8 ± 2.4	230.9 ± 3.6	268.7 ± 6.9
Control	58.1 ± 0.9	87.5 ± 1.4	132.5 ± 5.3	179.8 ± 3.0	231.7 ± 9.6	261.8 ± 6.8
Probiotics	58.7 ± 1.5	86.8 ± 2.8	135.0 ± 6.5	180.6 ± 6.2	228.1 ± 7.9	266.5 ± 11.4
Antagonist	57.4 ± 1.5	86.0 ± 2.8	132.9 ± 3.8	181.3 ± 3.8	228.1 ± 7.9	263.3 ± 4.7

### Oral strain colonisation in WISTAR rats

**Fig. 3:**
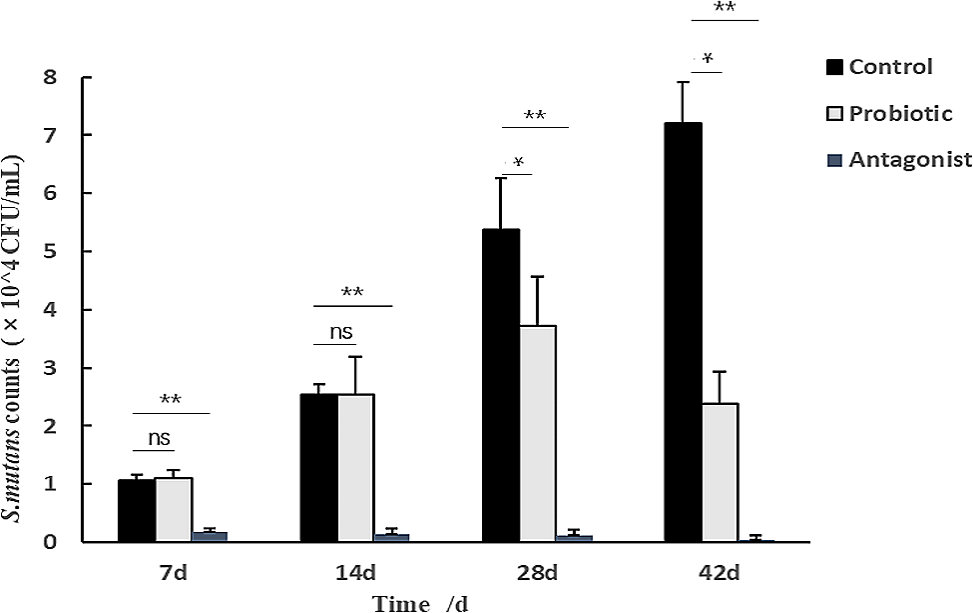
Counts of *S. mutans* in the oral cavity of each group of rats. *P < 0.05, compared with the control group; ** *p* < 0.01, compared with the control group; ns, not significant

Bacterial colonization of the oral cavity of each group of rats was performed on days 7, 14, 28, and 42 (Fig. 3). The rats in the N (normal) group did not contain *S. mutans*. The number of *S. mutans* in the C (caries control) and P (probiotic) groups gradually increased over 7 days, showing stable colonization. The concentrations of *S. mutans* on the molars of the C and P groups was approximately 1.0 × 10^4^ CFU/mL after 7 days of infection, indicating that *S. mutans* had successfully colonized the oral cavities. In the C group, the amount of *S. mutans* increased with feeding extension time and reached 7.2 × 10^4^ CFU/mL at 42 days. In the P group, the amount of *S. mutans* increased first and then decreased with the intervention of probiotics. At 42 days, the amount of *S. mutans* in the P group was 0.4 times that in the C group, and the number significantly decreased (*p* < 0.05). In the A (antagonist) group, the number of *S.mutans* remained low throughout the trial period, about 0.5–1.7 × 10^3^ CFU/mL. The amount of *S. mutans* in the A group was low and highly significant compared with the C group (*p* < 0.01).

### Observation and scoring of the dental profile of rats

**Fig. 4:**
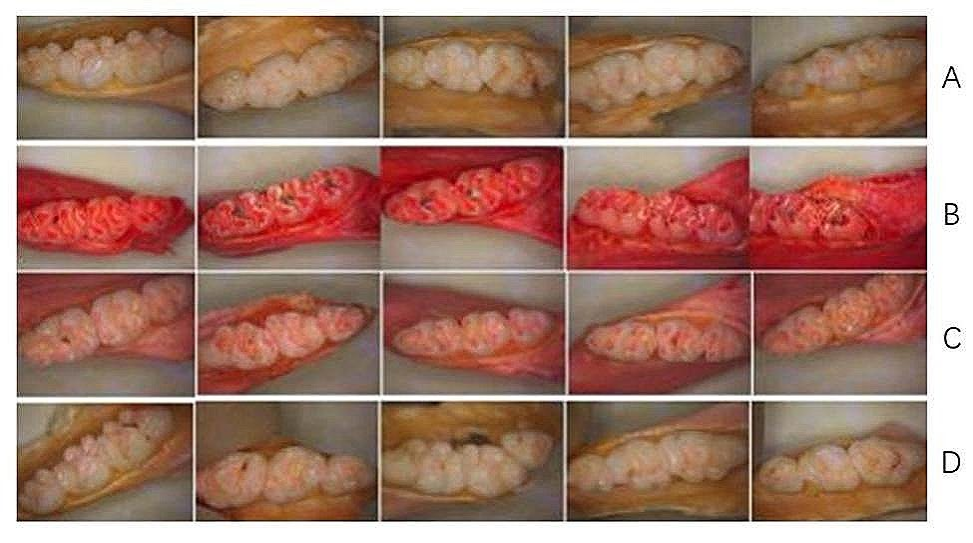
Results of ammonium violet urethane staining of rat teeth. (**A**) Normal; (**B**) Caries control; (**C**) Probiotic; (**D**) Antagonist

After the rat jaw bone samples were stained with ammonium purpurate staining solution, we observed that the molar teeth of the C group had different degrees of red stained caries areas under the in vivo microscope (Zeiss P30020001, Germany) (Fig. 4). The red-stained areas were observed in both the profile and cross-sectional views, indicating that the model was successfully constructed (Fig. 4B). This model can build a stable and qualified scheme for the pharmacodynamic evaluation of dental caries.

Figure 4C shows that the cavity scores were significantly reduced in the P group. Figure 4A and D shows that there was no evidence of dental caries visually in the A and N groups. The cheek, tongue and adjacent surfaces, and sulcal of a rat molar can be divided into several dental caries units according to the extent of caries damage, as shown in Table 2. Furthermore, Table 2 demonstrates how the dental caries units were determined and scored; the score results for the corresponding caries are shown in Table 4. According to the Keyes scoring rules, 12 maxillary and mandibular molars from each group were scored on 4 dimensions: E, Ds, Dm, and Dx. Caries scores for all 4 dimensions (i.e., E, Ds, Dm, and Dx) were significantly (*p* < 0.05) lower in the P-administered group compared with the C group. Moreover, caries scores on the sulcus surface in the A group were highly significant lower compared with the C group (*p* < 0.01). These caries scores in the P and A groups were less than in the C group, suggesting that probiotics have the potential to prevent caries in vivo.

**Table 4:** Effect of different groups on caries development (incidence and severity) in rats

Group	Caries level			
	E	Ds	Dm	Dx
**Control**	30.4 ± 3.72	20.2 ± 5.27	8.8 ± 2.04	3.0 ± 2.1
**Probiotics**	24.8 ± 2.04*	14.8 ± 0.98*	5.2 ± 0.98**	0.8 ± 0.75*
**Antagonism**	1.2 ± 0.75**	-	-	-

Dm, moderate dentinal penetration; Ds, slight dentinal penetration; Dx, extensive dentinal penetration; E, enamel only

*p < 0.05 compared with the control group; **p < 0.01 compared with the control group

### WISTAR Rat teeth grinding X-ray images

**Fig. 5:**
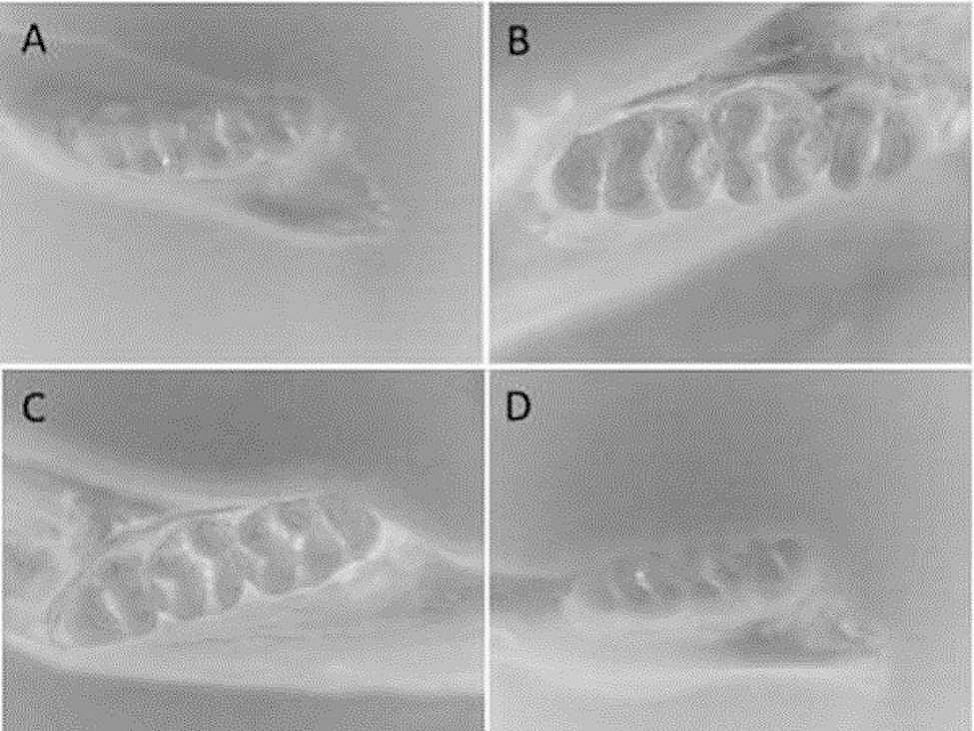
The X-ray results of the rat molars. (**A**) Normal; (**B**) Caries control; (**C**) Probiotic; (**D**) Antagonist

The caries status of the molars of rats in each group was determined by X-ray diffraction. The results are shown in Fig. 5. The alveolar bone of the rats in all groups was well aligned, with no obvious abnormalities and no significant widening of the periodontal gaps. In the C and P groups, a certain degree of bone loss was observed in the alveolar bone (Fig. 5B, C). No alveolar damage was observed in the N and A groups (Fig. 5A, D). These 4 strains of probiotics had some protective effects on the molar tissue of rats.

## Discussion

Dental caries is a multifactorial disease. Its pathogenesis involves the microbiota (mainly *S. mutans*), the host, and its immune mechanisms and diet, among other factors [32–34]. Even though all these factors must intervene for dental caries to develop, microbiological factors are still the leading cause of this disease. Therefore, bacteriotherapy is believed to positively influence the prevention of this disease [33–36].

*S. mutans* forms plaque biofilms through surface protein binding to sites on acquired membranes on the tooth surface. Biofilms are the main cause of dental caries formation. Therefore, the inhibition and removal of dental plaque biofilm are important for preventing caries [37]. In-vitro studies suggest that probiotics can prevent the adhesion of *S. mutans* to saliva-coated hydroxyapatite and interfere with caries-related biofilm formation [38–41]. The inhibitory effects of probiotics against *S. mutans* may be due to antimicrobial substances produced by probiotics, including organic acids, hydrogen peroxide, bacteriolytic enzymes, bacteriocins, and biosurfactants [42, 43]. In this study, the probiotics mixture showed good inhibition of the growth of *S. mutans* under both dynamic conditions (co-culture) and static conditions (inhibition test). Based on these results, it is hypothesized that these lactic acid bacteria can metabolize antibacterial substances, such as lactic acid and antimicrobial peptides. Organic acids can reduce the pH of the environment, thus inhibiting the growth of pathogenic bacteria. Antimicrobial peptides can interfere with the synthesis of bacterial cell wall of pathogenic bacteria, so as to achieve antibacterial effect. The whole genomes of these four strains have been uploaded into the NCBI database (M14, CP095384; OF10, CP092498; V38, CP092501; A17, CP097165), and they can also be found to have bacteriocin-related coding genes. Both the supernatant and the bacteria were able to remove the biofilm of *S. mutans*. The removal of biofilm by supernatant may be the effect of acid, which causes *S. mutans* to fall off the chamber slides. The removal of the biofilm by the bacteria should be that the probiotics have recognition protein, which can bind to the recognition protein of *S. mutans*, thereby destroying the biofilm. The inhibitory effects of *Lactobacillus* on *S. mutans* in vitro do not necessarily mean that *Lactobacillus* has the same effects in vivo. Therefore, this study further examined the anti-caries activity of the 4 strains in a rat model of dental caries. In the first 14 days of the rat experiment, there was no significant difference in the number of *S. mutans* between the control group and the probiotic group, indicating that the short intervention of probiotics did not play an inhibitory role. In the 28–42 days of intervention, the bacteria amount of *S. mutans* in probiotics was significantly lower than that in the control group, indicating that probiotics could inhibit the growth of *S. mutans* with the extension of intervention time (Fig. 3). But probiotics don’t completely eliminate *S. mutans*. From the score results and pictures (Figs. 4 and 5), the cavity scores were reduced after probiotic prevention. These results indicate that the 4 strains may be useful in the prevention of dental caries caused by *S. mutans*. The antagonistic experimental group simulates the coexistence of lactic acid bacteria and *S. mutans* in the mouth. Probiotics and *S. mutans* were applied to the teeth of rats at the same time to see how the teeth changed. The results showed that there was only a small amount of *S. mutans* on the teeth of rats in the antagonistic group on the 7th day, and basically no streptococcus mutans growth in the later period. From the staining and X-ray tooth pictures (Figs. 4 and 5), the appearance of teeth in the antagonistic group was no different from that in the normal group, indicating that taking probiotics could prevent the occurrence of dental caries in rats without dental caries. These results indicate that the 4 strains may be contribute to the prevented the development of dental caries.

The clinical effectiveness of probiotics depends on the appropriate strain, duration of treatment, concentration, and the ideal vehicle for use. Current studies have shown that probiotic products can reduce the population of *S. mutans* in the human oral cavity and could have a preventive effect on the development of dental caries [34, 44–47]. Future studies will include clinical trials at a later stage to explore the clinical performance of these 4 bacterial strains and lay the foundation for possible product development. Interest in postbiotics is increasing as there are limitations to the storage and use of probiotics [48]. Considering the potential future application areas, we will also explore the clinical efficacy of the postbiotic of this complex probiotic at a later stage.

## Data Availability

The data presented in this study are available within the article.
